# miR-212-5p Regulates PM_2.5_-Induced Apoptosis by Targeting LAMC2 and LAMA3

**DOI:** 10.3390/ijms26041761

**Published:** 2025-02-19

**Authors:** Yunna Jia, Xiqing Zhang, Cuizhu Zhao, Zhenhua Ma, Ke Sun, Yize Sun, Xiaohui Du, Meng Liu, Xiaojun Liang, Xiuzhen Yu, Yunhang Gao

**Affiliations:** 1 Department of Veterinary Medicine, College of Animal Science and Technology, Jilin Agricultural University, Changchun 130118, China; 15590074644@163.com (Y.J.); zhangxiqing1020@163.com (X.Z.); zhaocuizhu2024@163.com (C.Z.); mazhenhua1030@163.com (Z.M.); sun155198@163.com (K.S.); sunyize1228@163.com (Y.S.); duxiaohui2001@163.com (X.D.); liumeng4610@163.com (M.L.); 2 Institute of Animal Science, Ningxia Academy of Agriculture and Forestry, Yinchuan 750002, China; lxj0520@163.com; 3 Institute of Agricultural Mechanisation, Xinjiang Academy of Agricultural Sciences, Wulumuqi 830091, China

**Keywords:** fine particulate matter, lung injury, miR-212-5p, LAMC2, LAMA3, apoptosis

## Abstract

Fine particulate matter (PM_2.5_) is often linked to a range of respiratory diseases and cellular damage. Although studies have shown that the expression profiles of microRNAs (miRNAs) are altered during lung damage brought on by PM_2.5_, the underlying functions of miRNAs remain poorly understood. In this research, we explored the role of PM_2.5_-induced apoptosis in detail and focused on the miRNA (miR-212-5p) that regulates apoptosis. Through a dual-luciferase assay, a direct targeting connection between laminin subunits γ2 (*LAMC2*) and α3 (*LAMA3*) and miR-212-5p was successfully demonstrated. This study focused on revealing the negative regulatory relationship between miR-212-5p and *LAMC2* and *LAMA3*, providing important clues for a deeper understanding of the relevant physiological and pathological mechanisms. The present study showed that LAMC2 and LAMA3 positively regulate the PI3K-AKT pathway and negatively regulate the NF-κB pathway, which directly leads to significant changes in apoptosis rates. This study reveals a previously unrecognized molecular mechanism by showing that miR-212-5p directly targets *LAMC2* and *LAMA3* and thus associates with PM_2.5_-induced apoptosis via the PI3K/AKT/NF-κB pathway. These findings not only redefine the role of miR-212-5p in apoptosis but also open up new avenues for future research.

## 1. Introduction

One of the main pollutants used to measure indoor air quality is particulate matter 2.5 (PM_2.5_), which comprises particles of matter with a diameter of less than 2.5 μm [1]. There are multiple ways for PM_2.5_ to enter an animal’s body, including inhalation, skin contact, and conjunctival exposure, and it can spread rapidly throughout the body via the bloodstream [2,3]. Reactive oxygen species (ROS) are produced in huge quantities in cells due to extended encounters with high levels of PM_2.5_ in the body, according to numerous scientific studies, and they induce oxidative stress and activate downstream proteases such as cysteine caspase-3 (caspase-3), a key protease for apoptosis, ultimately leading to cell apoptosis [4,5,6]. In recent years, due to the widespread use of large-scale intensive farming models [7], indoor concentrations of PM_2.5_ on farms have far exceeded those in urban areas, and emissions of polluted air from livestock buildings to the outside are causing ecosystem changes and even endangering the health of farm workers [8,9]. Determining the particular mode of action of PM_2.5_-induced apoptosis in the respiratory tract is therefore particularly important for providing new therapeutic targets for the prevention of lung injury.

Being approximately 20–24 nucleotides long, miRNAs are a type of endogenous short RNA that do not directly encode proteins but are involved in everything from basic biological processes, such as the regulation of apoptosis, to complex physiological processes [10]. There are studies that link miRNAs to PM_2.5_-induced cell damage. For example, PM_2.5_-induced asthmatic airway epithelial barrier breakdown is promoted by miR-129-2-3p [11]. miR-421 affects PM_2.5_-induced endothelial dysfunction [12]. However, as PM_2.5_ continues to erode lung health and trigger lung damage, there is essentially a gap in the research on the role miRNAs play in alveolar macrophage apoptosis and the mechanisms involved. In addition, abnormal binding of miRNAs to target genes is a crucial factor in many significant diseases [2]. By detecting changes in the binding status of certain specific miRNAs to target genes, they can be used as markers for early diagnosis of disease. However, miRNAs are not unique in their target mRNAs, which means that therapies targeting miRNAs can have complex and unpredictable side effects [13]. On the other hand, the precise regulation of miRNA interactions with multiple target genes makes it possible to intervene in several pathological aspects of the disease at the same time and improve therapeutic efficacy. As a result, identifying miRNA target genes and creating an miRNA target gene network are especially crucial.

An essential part of the extracellular matrix (ECM) is the laminin (LN) protein family [3]. An intact LN protein has three peptide chains, of which the α chain has five isoforms (α1, α2, α3, α4, α5), the β chain has four isoforms (β1, β2, β3, β4), and the γ chain has three isoforms (γ1, γ2, γ3), and combinations of the different isoforms of α, β, and γ form a cross-like structure of the heterotrimer [14]. By binding to cell surface receptors, laminin activates signaling pathways such as NF-κB, increases anti-apoptotic gene expression, and inhibits the activation of the caspase family to escape apoptosis. It has been shown that *LAMC2* and *LAMA3*, when used as target genes of miRNAs, participate in apoptosis and other cellular damaging processes; for example, LAMA3 and LAMC2 are involved in encoding laminin 332, which, in pancreatic ductal adenocarcinoma, causes apoptosis and the epithelial–mesenchymal transition [15]. Normal expression of LAMC2 and LAMA3 contributes to the orderly arrangement and functionality of tissue cells and avoids unwanted apoptosis, but the role of miRNAs in this approach is unclear.

The aim of this study was to elucidate the molecular mechanism of PM_2.5_-induced apoptosis. First, we analyzed PM_2.5_-exposed samples to screen for differentially expressed miRNA genes and finally identified miR-212-5p as the key research target. *LAMC2* and *LAMA3* were confirmed as miR-212-5p target genes via a dual-luciferase reporter system. Based on this, we further investigated the process of PM_2.5_-induced apoptosis and confirmed that LAMC2 and LAMA3 were involved in PM_2.5_-induced apoptosis through the PI3K/AKT/NF-κB pathway. These outcomes enrich the target gene regulatory network of miR-212-5p and may provide novel targets for the treatment of lung injury.

## 2. Results

### 2.1. PM_2.5_ Caused NR8383 Cell Activity to Decline

**Figure 1:**
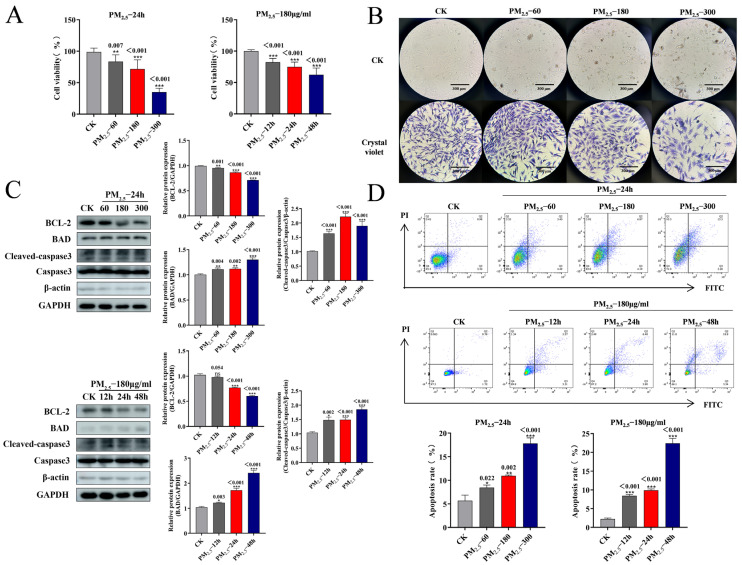
PM_2.5_-induced decreased cell activity and apoptosis in NR8383 cells: (**A**) CCK-8 detection of cell viability; (**B**) morphological changes in NR8383 after PM_2.5_ stimulation; (**C**) Western blotting detection of BCL-2, BAD, and caspase-3 protein changes after PM_2.5_ stimulation; (**D**) flow cytometry detection of apoptosis rate after PM_2.5_ stimulation. The mean ± standard deviation (*n* = 3) is used to express the data. ns, no significance; *, *p* < 0.05; **, *p* < 0.01; and ***, *p* < 0.001 indicate significance.

We studied the cell viability of NR8383 stimulated by varying PM_2.5_ concentrations or stimulated by the same PM_2.5_ concentration for varying durations to determine PM_2.5_’s effects on NR8383 cell viability. According to the CCK8 data, cell viability was considerably decreased by PM_2.5_ doses of 60 μg/mL, 180 μg/mL, and 300 μg/mL. As illustrated in Figure 1A, the cell viability of NR8383 was significantly reduced after 12, 24, and 48 h of stimulation with PM_2.5_ values of 180 μg/mL. Changes in cell morphology were observed at 400× magnification. Compared with the CK group, the morphology of NR8383 cells in the PM_2.5_-exposed group shifted from a shuttle shape with pseudopods to a spindle shape, and the cells became rounded, with coalesced and clumped chromatin and crumpled cytoplasm (Figure 1B).

### 2.2. NR8383 Cell Apoptosis Brought on by PM_2.5_

We know that PM_2.5_ can lead to a variety of cellular damage, and apoptosis is one of the major types of damage. To establish how PM_2.5_ affects apoptosis in NR8383 cells, we examined BCL-2, BAD, and caspase-3 protein expression in NR8383 cells stimulated with PM_2.5_. As shown in Figure 1C, the WB results show that BAD and cleaved caspase-3 expression gradually increased and BCL-2 expression gradually decreased under the effect of 60 μg/mL, 180 μg/mL, and 300 μg/mL PM_2.5_. NR8383 was stimulated with 180 μg/mL PM_2.5_ for 12, 24, and 48 h. After NR8383 stimulation at 180 μg/mL PM_2.5_ for 12 h, 24 h, and 48 h, BAD and cleaved caspase-3 expression gradually increased, and BCL-2 gradually decreased (Figure 1C). The apoptosis rate of NR8383 gradually increased as PM_2.5_ stimulation concentration or the prolongation of the action time of the same concentration increased, as detected with flow cytometry (Figure 1D). It was concluded that apoptosis could be brought on by PM_2.5_ of NR8383 cells, and the apoptosis showed a significant increase as PM_2.5_ stimulation concentration or the prolongation of the effect time of the same concentration increased.

### 2.3. miR-212-5p Promotes PM_2.5_-Induced Apoptosis in NR8383 Cells

**Figure 2:**
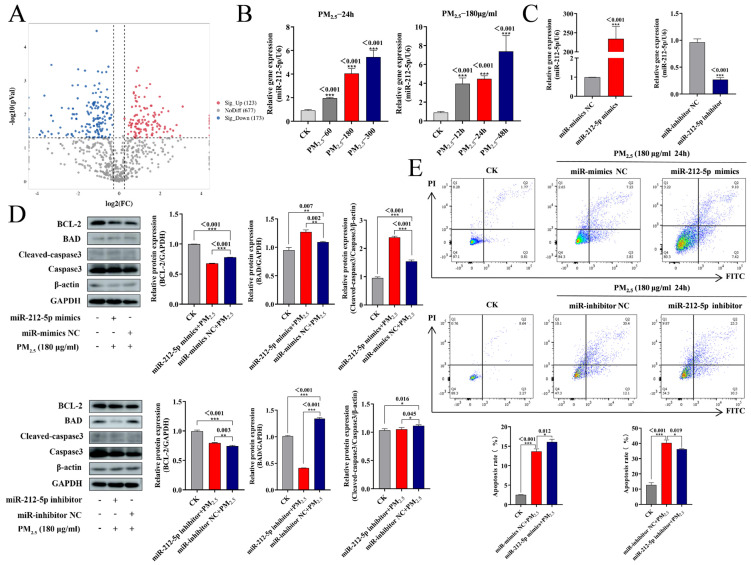
miR-212-5p promotes NR8383 cell apoptosis brought on by PM_2.5_: (**A**) volcano plot of miRNAs in rat lung after PM_2.5_ stimulation; (**B**) expression variations in miR-212-5p detected with RT-PCR after PM_2.5_ stimulation; (**C**) transfection efficiency of miR-212-5p mimics and miR-212-5p inhibitors detected with RT-PCR; (**D**) transfection of miR-212-5p mimics or miR-212-5p inhibitors under PM_2.5_ stimulation (180 μg/mL for 24 h), and changes in BCL-2, BAD, and caspase-3 proteins detected with Western blotting; (**E**) apoptosis rate detected with flow cytometry. The mean ± standard deviation (*n* = 3) is used to express the data. ns, no significance; *, *p* < 0.05; **, *p* < 0.01; and ***, *p* < 0.001 indicate significance.

To investigate whether miRNAs are involved in PM_2.5_-induced NR8383 apoptosis, we observed the expression of miRNAs in rat lungs after PM_2.5_ stimulation. miRNAs appeared to be significantly altered in the PM_2.5_-exposed group compared with the CK group, with 296 differential miRNAs (Figure 2A). After further screening for differentially expressed miRNAs, miR-212-5p caught our attention, with miR-212-5p gene expression being greatly increased in the PM_2.5_-exposed group compared to the CK group at all PM_2.5_ concentrations and exposure times (Figure 2B). It is known that miRNA mimics increase the expression of mature miRNAs in cells, whereas miRNA inhibitors have the opposite outcome. We transfected miRNA mimics (miR-212-5p mimics) and inhibitors (miR-212-5p inhibitors) into NR8383 cells. According to Figure 2C, miR-212-5p expression was increased 234-fold in NR8383 cells transfected with miR-212-5p mimics, whereas transfection with miR-212-5p inhibitor decreased miR-212-5p expression by more than 70%. The effect of miR-212-5p on apoptotic gene expression was examined further, and as shown in Figure 2D, under the influence of 180 μg/mL PM_2.5_, the expression of BCL-2 was reduced while that of BAD was elevated, and cleaved caspase-3 was increased, whereas the action of the miR-212-5p inhibitor reversed these manifestations, which were further enhanced when miR-212-5p mimics were present. Flow cytometry detection of the apoptosis rate demonstrated that PM_2.5_ increased the apoptosis rate of NR8383 cells compared to CK, and it was further increased after transfection with miR-212-5p mimics. Transfection with an miR-212-5p inhibitor reduced the rate of apoptosis in comparison to the PM_2.5_ group (Figure 2E). These findings imply that miR-212-5p positively regulates NR8383 cell apoptosis brought on by PM_2.5_.

### 2.4. miR-212-5p Negatively Regulates LAMC2 and LAMA3

**Figure 3:**
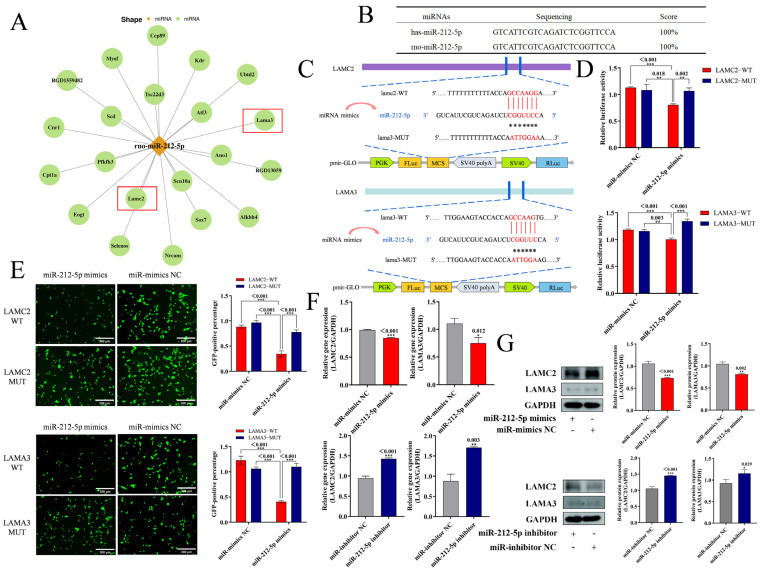
miR-212-5p negatively regulates LAMC2 and LAMA3: (**A**) miRanda (v3.3a) and TargetScan (v5.0) projected miR-212-5p target genes displayed; (**B**) similarity of rno-miR-212-5p and has-miR-212-5p genes shown; (**C**) schematic representation of LAMC2-3′ UTR and LAMA3-3′ UTR with miR-212-5p target prediction and reporter plasmid construction, where red characters indicate binding sites and ”*” characters indicate mutation sites; (**D**) luciferase activity assessed in 293T cells after co-transfection of LAMC2-WT, LAMC2-MUT, LAMA3-WT, and LAMA3-MUT with miR-212-5p mimics or miR-mimics NC for 48 h; (**E**) LAMC2-mVenus-WT, LAMC2-mVenus-MUT, LAMA3-mVenus-WT, and LAMA3-mVenus-MUT co-transfected with miR-mimics NC or miR-212-5p in 293T cells (scale bar: 300 µm); (**F**) RT-PCR assay for miR-212-5p mimics and miR-212-5p inhibitors on *LAMC2* and *LAMA3* expression after transfection; (**G**) Western blotting used to identify alterations in the LAMC2 and LAMA3 proteins. The mean ± standard deviation (*n* = 3) is used to express the data. ns, no significance; *, *p* < 0.05; **, *p* < 0.01; and ***, *p* < 0.001 indicate significance.

To explore the specific mechanism of miR-212-5p involvement in NR8383 apoptosis, we predicted miR-212-5p downstream target genes using MiRanda (v3.3a) and Targetscan (v5.0), among which the laminin family protein genes LAMC2 and LAMA3 both have the potential to bind to miR-212-5p (Figure 3A), in addition to analyzing the rat-derived miR-212-5p (rno-miR-212-5p) and human-derived miR-212-5p (has-miR-212-5p) genes with 100% similarity and homology (Figure 3B). To verify the connection between miR-212-5p and *LAMC2* and *LAMA3*, we generated wild-type (WT) and mutant (MUT) plasmids with LAMC2-3′ UTR sequences as well as WT and MUT plasmids with LAMA3-3′ UTR sequences (Figure 3C). Firefly luciferase activity was markedly decreased in the co-transfection group by miR-212-5p and the WT plasmid (Figure 3D). Binding was observed using fluorescence intensity, and the co-transfected group of miR-212-5p with the wild-type plasmid suppressed the expression of the mVenus fluorescent protein (Figure 3E). These outcomes demonstrated that miR-212-5p directly targets *LAMC2* and *LAMA3*, with the 3′ UTR of *LAMC2* and *LAMA3* serving as their respective target binding sites. *LAMC2* and *LAMA3* expression was then detected using miR-212-5p mimics and miR-212-5p inhibitors, respectively, after transfection of NR8383 cells. *LAMC2* and *LAMA3* gene expression could be considerably down-regulated by miR-212-5p mimics and elevated by the miR-212-5p inhibitor, according to the RT-qPCR results (Figure 3F). The WB test results also agreed with the qPCR results (Figure 3G). In conclusion, we found that miR-212-5p could target *LAMC2* and *LAMA3*, and miR-212-5p showed a negative regulatory relationship with LAMC2 and LAMA3.

### 2.5. PM_2.5_-Induced Changes in LAMC2 and LAMA3 Expression

**Figure 4:**
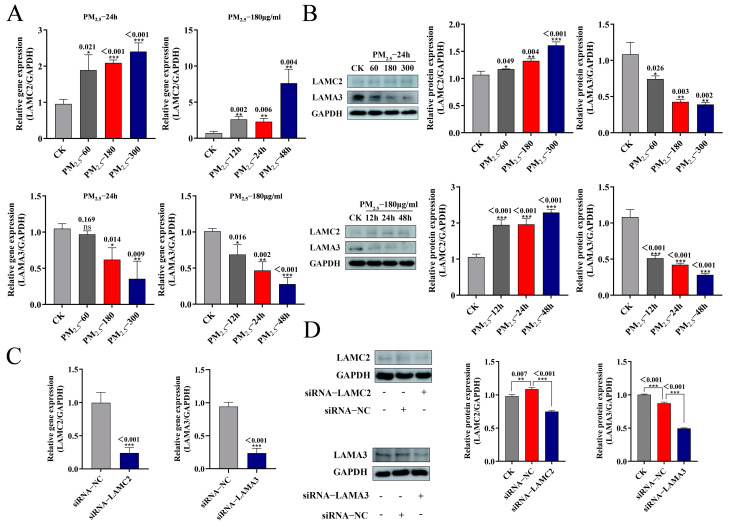
PM_2.5_-induced changes in LAMC2 and LAMA3 expression: (**A**) LAMC2 and LAMA3 gene expression detected with RT-PCR after PM_2.5_ stimulation; (**B**) LAMC2 and LAMA3 protein changes detected with Western blotting; (**C**) silencing efficiency of LAMC2 and LAMA3 after transfection with siRNA−LAMC2 or siRNA−LAMA3 detected with RT-PCR; (**D**) LAMC2 and LAMA3 protein changes detected with Western blotting. The mean ± standard deviation (*n* = 3) is used to express the data. ns, no significance; *, *p* < 0.05; **, *p* < 0.01; and ***, *p* < 0.001 indicate significance.

Though additional confirmation of the precise functions of the target genes LAMC2 and LAMA3 in this process is still required, the foregoing data show that miR-212-5p was implicated in PM_2.5_-induced apoptosis in NR8383 cells. Initially, we looked at the expression of LAMC2 and LAMA3 in NR8383 driven by PM_2.5_. The findings indicate that in contrast to CK, LAMC2 expression tended to increase and LAMA3 expression tended to decrease with the rise in PM_2.5_-stimulated concentration or the prolongation of the action time of the same concentration, both at the gene level detected with RT-qPCR (Figure 4A) and at the protein level detected with WB (Figure 4B). These findings imply that PM_2.5_-induced apoptosis may be regulated by LAMC2 and LAMA3.

### 2.6. LAMC2 and LAMA3 Inhibit Apoptosis via the PI3K/AKT/NF-κB Pathway

**Figure 5:**
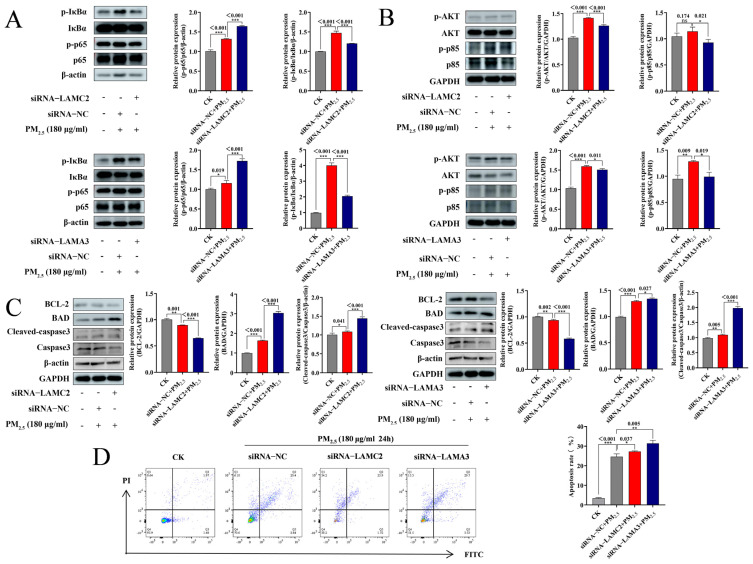
LAMC2 and LAMA3 inhibit apoptosis through the PI3K/AKT/NF-κB pathway: (**A**) PM_2.5_-stimulated (180 μg/mL for 24 h) cells transfected with siRNA−LAMC2 or siRNA−LAMA3, and p-IκBα/IκBα and p-p65/p65 protein changes; (**B**) p-AKT/AKT and p-p85/p85 protein changes; (**C**) BCL-2, BAD, and caspase-3 protein changes detected with Western blotting; (**D**) apoptosis rate detected with flow cytometry. The mean ± standard deviation (*n* = 3) is used to express the data. ns, no significance; *, *p* < 0.05; **, *p* < 0.01; and ***, *p* < 0.001 indicate significance.

To further investigate the possible involvement of LAMC2 and LAMA3 in PM_2.5_-induced apoptosis, siRNAs (siRNA−LAMC2, siRNA−LAMA3) were designed against *LAMC2* and *LAMA3*, respectively, to further validate the effects of LAMC2 and LAMA3 on the NF-κB signaling pathway. According to Figure 4C, siRNA−LAMC2 transfection significantly suppressed the gene expression of *LAMC2*. The results of WB detection of LAMC2 and LAMA3 protein expression were consistent with the trend of RT-qPCR (Figure 4D). While p-p65 expression increased p-IκBα expression under PM_2.5_ stimulation, the NF-κB pathway was activated. p-IκBα/IκBα expression decreased and p-p65/p65 expression increased in the presence of siRNA−LAMC2 or siRNA−LAMA3, indicating that the NF-κB pathway was further triggered (Figure 5A). In addition, the PI3K/AKT pathway could also regulate apoptosis by phosphorylating BAD, as shown in Figure 5B. While the PI3K/AKT pathway was triggered by PM_2.5_ stimulation, this phenomenon could be reversed in the presence of siRNA−LAMC2 or siRNA−LAMA3. Following PM_2.5_ exposure, the BCL-2 expression decreased; BAD and cleaved caspase-3 were further increased, and BCL-2 was further decreased in the presence of siRNA−LAMC2 or siRNA−LAMA3 (Figure 5C). These results suggest that either LAMC2 or LAMA3 can inhibit apoptosis of NR8383 cells through the PI3K/AKT/NF-κB pathway.

## 3. Discussion

In recent years, the intensive and large-scale development of livestock and poultry farming has contributed to the continuous increase in PM_2.5_ levels in the agricultural environment, which greatly increases the probability of animals suffering from respiratory diseases, and various types of cellular damage, such as apoptosis, are involved in the process of developing respiratory conditions [16]. Many studies have shown that miRNAs, a type of non-coding RNA, may participate in controlling biological processes such as apoptosis. Although it has been shown that PM_2.5_ can interfere with intracellular signaling pathways, which in turn affects the expression of miRNAs, there is still a relative lack of research on the specific mechanisms by which miRNAs affect lung injury [17,18]. In this study, we first analyzed the miRNAs after PM_2.5_ stimulation and found that 296 miRNAs, including miR-212-5p, were differentially expressed, and then we carried out a profound exploration based on the function of PM_2.5_-induced apoptosis and identified miRNAs that adjust apoptosis (miR-212-5p). The first molecular mechanism of miR-212-5p binding to *LAMA3* and *LAMC2* to regulate apoptosis in NR8383 cells via the PI3K/AKT/NF-κB pathway has been revealed.

Differences in the concentration and composition of PM_2.5_ in different environments affect its biological toxicity [19]. Some researchers extracted PM_2.5_ whole particles, water-soluble extracts, organic extracts, and carbon core components and found that PM_2.5_ whole particles and a mixture of PM_2.5_ whole particles and Po caused the most severe cell damage [20]. Furthermore, high concentrations of PM_2.5_ are more likely to cause damage such as apoptosis, but long-term exposure to low concentrations of PM_2.5_ instead promotes cell proliferation, with a two-way effect [21]. Cell proliferation has been shown to be enhanced when cells are exposed to concentrations below 100 μg/mL PM_2.5_ for long periods of time. PM_2.5_ at 60 μg/mL caused a decrease in cell viability and apoptosis in this study, which is related to the different cell lines we used. In this study, alveolar macrophages were selected to phagocytose particulate matter and pathogens in the lungs, and apoptosis was also induced by the adsorption of larger particles on the surface of macrophages that could not be engulfed by the cells [22]. This was confirmed to be due to the activation of mitochondrial autophagy through the ROS/AKT/FOXO1 pathway, which promotes ROS production [23]. It has also been shown that particulate matter-induced damage can occur in multi-organ cascades, such as damage to brain tissue via the liver–brain axis [24]. The different sources of agricultural PM_2.5_ that we used carry more pathogenic microorganisms compared to urban PM_2.5_ [25,26].

There are many potential gene therapy targets that can be used to explore the process of inhibiting the biological toxicity of PM_2.5_ with biologics, and the role of miRNAs in this complex process is becoming increasingly emphasized. Inside the cell, miRNAs first form the core reduced instruction set computer (RISC) assembly with argonaute protein (AGO) and then pair complementarily with the 3′ UTR of the target mRNA through its seed sequence, which can be either fully or partially complementary [27]. Four distinct mechanisms are believed to be involved in in vivo miRNA translation repression: mRNA degradation, premature translation termination, inhibition of translation initiation, and inhibition of translation elongation [28]. By suppressing SUMO2 expression, miR-212-5p has been demonstrated to control glioma cell migration, hyperplasia, and apoptosis. As such, in the treatment of gliomas, it might be a promising therapeutic target [29]. Similar results have been seen in many tumors, such as colorectal and nasopharyngeal cancer, where miR-212-5p prevents additional cancer growth by inhibiting target gene expression [30,31]. In this study, we predicted and analyzed the mRNAs affected by miR-212-5p and established that *LAMC2* and *LAMA3* are the target genes of miR-212-5p, which controls their transcriptional expression.

miRNAs are characterized by the fact that their target mRNAs do not have a single counterpart, a feature that makes miRNA-based therapeutic strategies highly likely to cause unpredictable side effects. For example, trying to treat cancer by inhibiting miR-155 may interfere with normal immune regulation because it also acts on immune-related target genes [32]. On the other hand, this multi-targeting property also offers the opportunity to develop comprehensive therapeutic strategies. For example, miR-200 is emerging as a novel star miRNA by targeting the transcription factors ZEB1 and ZEB2 to inhibit EMT and thereby prevent further development of malignant tumors [33]. Similarly, this study showed that miR-212-5p targets *LAMC2* and *LAMA3*, and both genes are involved in apoptosis, which enhances the miR-212-5p target genes’ regulatory network but also improves the therapeutic effect. On the other hand, this study compared the highly conserved sequences of rno-miR-212-5p and has-miR-212-5p, which greatly facilitates cross-species studies.

Interestingly, in our study, LAMC2 and LAMA3, although both laminin-family genes, showed opposite changes in NR8383 cells after PM_2.5_ exposure, with increased LAMC2 expression and decreased LAMA3 expression after PM_2.5_ stimulation. Related studies suggest that differences in LAMC2 and LAMA3 expression after PM_2.5_ stimulation are perhaps connected to PM_2.5_-induced changes in cell migration ability [34]. When the expression of LAMC2 and LAMA3 is increased, intercellular adhesion is weakened, resulting in the detachment of cells from one site to colonize other sites and the loosening of intercellular junctions accompanied by an increased ability to migrate [35]. However, it is clear that both LAMC2 and LAMA3 can regulate PM_2.5_-induced apoptosis.

To date, miR-212-5p has been shown to target *LAMC2* and *LAMA3* to regulate apoptosis caused by PM_2.5_. However, because of the diverse functions of miRNAs—for example, pri-miRNAs can encode regulatory peptides [36], and miRNAs can act as agonists to activate toll-like receptors [37]—the functional exploration of miR-212-5p is incomplete, and the possibility that miR-212-5p has the ability to regulate positively or negatively throughout the process of host injury has not been ruled out [38]. Thus, further research is needed in the future to shed light on participation in the miR-212-5p/mRNA regulatory axis in organisms.

## 4. Materials and Methods

### 4.1. PM_2.5_ Preparation

As described in a previous study [39], we collected PM_2.5_ in a barn using a TPS atmospheric sampler (Lonying Environmental Technology Co., Qingdao, China) and analyzed its main chemical composition and characterization [40], which was finally formulated into a 10,000 μg/mL solution and stored.

### 4.2. miRNA Mimics, Inhibitors, and RNA Interference

miR-212-5p mimics in line with negative controls (miR-mimics NC), miR-212-5p inhibitors in line with negative controls (miR-inhibitor NC), and siRNAs for *LAMC2* and *LAMA3* (siRNA−LAMC2, siRNA−LAMA3) were obtained from GenePharma (Suzhou, China), and details are displayed in Table S1. The siRNAs, mimics, or inhibitors and their respective negative controls were transfected into NR8383 cells. We applied an siRNA/miRNA transfection chemical (Polyplus-transfection, Illkirch, France) in compliance with the manufacturer’s guidelines, with each transfection resulting in a final RNA concentration of 50 pmol/mL. miR-212-5p inhibitors, miR-212-5p mimics, siRNA−LAMC2, and siRNA−LAMA3 were detected using qPCR for efficiency. For every experiment, three biological replicates were planned.

### 4.3. Establishment of Animal and Cell Models

In this experiment, six-week-old SD rats with SPF (Changsheng Biotechnology, Benxi, China) were subjected to a PM_2.5_ exposure test using the tracheal drop method (Animal Experimentation Ethics Number: 20231206001) [41]. The test group (*n* = 5) was injected with a 50 μL volume of drop solution and 20 mg/kg of PM_2.5_.

Cell Bank provided the human kidney epithelial cell line (293T) and the rat alveolar macrophage cell line (NR8383) (Shanghai Institutes for Biological Sciences, Shanghai, China). At 37 °C in an incubator with 5% CO_2_, both cell lines were cultivated in high-sugar DMEM (Gibco, Grand Island, NY, USA) supplemented with 10% FBS, 100 U/mL penicillin, and 0.1 mg/mL streptomycin (Beyotime Biotechnology, Shanghai, China). The cells were passaged every 2 days at a ratio of 1:2. To evaluate the effects of different concentrations of PM_2.5_ exposure on NR8383, the cells in this study were separated into a blank group (CK) and groups with different concentrations of PM_2.5_ exposure (60 μg/mL, 180 μg/mL, or 300 μg/mL, 24 h). To assess the effect of different exposure times with the same concentration of PM_2.5_ on NR8383, cells were separated into CK and 180 μg/mL PM_2.5_ exposure (12 h, 24 h, 48 h) groups. To investigate the function of miR-212-5p during PM_2.5_ stimulation by NR8383, the cells were separated into CK, PM_2.5_ + miR mimic NC, and PM_2.5_ + miR-212-5p mimic, and CK, PM_2.5_ + miR inhibitor NC, and PM_2.5_ + miR-212-5p inhibitor. To further investigate the effects of LAMC2 and LAMA3 on the PI3K/AKT/NF-κB pathway, we silenced the LAMC2 and LAMA3 genes using siRNAs; categorized the cells into CK, PM_2.5_ + siRNA NC, PM_2.5_+siRNA−LAMC2, and PM_2.5_ + siRNA−LAMA3 groups; and detected the changes in the PI3K/AKT/NF-κB pathway.

### 4.4. Cell Viability

Cell viability was assessed using the Cell Counting Kit-8 (CCK-8) from Beyotime Biotechnology in Shanghai, China. A density of 5 × 10^3^ cells/well was used to inoculate 96-well plates with NR8383 cells. For every concentration, five biological replicates were exposed to 100 μL of PM_2.5_ at 60 μg/mL, 180 μg/mL, or 300 μg/mL. After that, 10 μL of CCK-8 were added to each well, which was then kept for two hours at 37 °C. The absorbance (OD_450_) was gauged.

### 4.5. Flow Cytometry

CK and PM_2.5_-exposed NR8383 were inoculated into 6-well plates, and cells were harvested with trypsin digestion (Gibco, Grand Island, NY, USA) at the end of exposure, the process was repeated using cooled PBS for washing, and the cells were counted. From each sample, 1 × 10^5^ cells were collected and resuspended with the dilution (BD Pharmingen^TM^, Franklin Lakes, NJ, USA), stained at room temperature for 15 min under light protection, and passed through a flow cytometer (BD LSRFortessa^TM^, BD Biosciences, Franklin Lakes, NJ, USA) within one hour. The distribution of apoptotic cells was analyzed using FlowJo 10, and all samples were analyzed using the same compensation and quadrants.

### 4.6. miRNA Microarray Assay

Three biological replicates were employed for the miRNA transcriptome expression analysis of the lungs of the rats in the PM_2.5_-exposed group and the rats in the CK group. To separate and purify total RNA, TRIZOL reagent (Invitrogen, Carlsbad, CA, USA) was utilized. Following the manufacturer’s instructions, a small RNA library was created using the Illumina Truseq Small RNA Preparation Kit (San Diego, CA, USA). ACGT101-miR (LC Sciences, Houston, TX, USA) was used to sequence the miRNA-seq reads. The t-test was utilized selectively to examine the variations in miRNA expression according to normalized deep sequencing counts, with a significant threshold of 0.05 for each test.

### 4.7. RNA Isolation and Quantitative Real-Time PCR

The integrity of the total RNA extracted from the cells (Sangon Biotech, Shanghai, China) was confirmed with agarose gel electrophoresis. mRNA was reverse-transcribed to cDNA (TaKaRa, Dalian, China). Utilizing TaKaRa’s (Dalian, China) kit, real-time quantitative PCR was carried out. The relative expression of the mRNA was adjusted to *GAPDH*. The miRNA was reverse-transcribed to cDNA (Vazyme, Nanjing, China), and quantitative PCR was performed in real time (Vazyme, Nanjing, China). *U6* was used to normalize the relative expression of miRNA. To evaluate miRNA expression levels, each group was given one pair of qPCR primers specific to miR-212-5p and *U6* as well as one RT primer. Table S2 contains a list of primers. Quantitative real-time PCR was carried out on a LightCycler 96 device (Roche, Basel, Switzerland). Relative mRNA or miRNA levels were calculated using formula 2^−(ΔΔCt)^.

### 4.8. miR-212-5p Target Gene Identification

Two algorithms, Targetscan (v5.0) [42] and MiRanda (v3.3a) [43], were utilized to forecast miR-212-5p target genes and their targets of action. Target genes were screened based on a comprehensive analysis of the number of complementary base pairs at the binding site, evolutionary conservation between species, predicted binding effect, and overall score.

### 4.9. Vector Construction

The LAMC2-3′UTR or LAMA3-3′UTR regions containing binding sites were ligated to the pmir-GLO plasmid (Miaolingbio, Wuhan, China) to construct wild-type dual-luciferase gene reporter plasmids (LAMC2-WT, LAMA3-WT) and ligated to the mVenus-C1 plasmid to build green fluorescent protein reporter plasmids (LAMC2-mVenus-WT, LAMA3-mVenus-WT). Mutant dual-luciferase gene reporter plasmids (LAMC2-MUT, LAMA3-MUT) and mutant green fluorescent protein reporter plasmids (LAMC2-mVenus-MUT, LAMA3-mVenus-MUT) were produced using point-specific mutation primers and a Vazyme kit (Vazyme, Nanjing, China). By extracting the plasmids (Tiangen, Beijing, China) after Sanger sequencing, we confirmed accurate synthesis. Table S3 displays the primer sequences.

### 4.10. Dual-Luciferase Reporter Assays

We performed co-transfection (Lipofectamine 3000, Thermo Fisher Scientific, Waltham, MA, USA) of miR-212-5p mimics or miR-mimics NC with wild-type dual-luciferase reporter plasmids (LAMC2-WT, LAMA3-WT) or mutant dual-luciferase reporter plasmids (LAMC2-MUT, LAMA3-MUT) into 293T cells. The transfection efficiency was then standardized using the sea kidney luciferase activity.

### 4.11. Western Blotting

Cells were lysed using RIPA lysis buffer (Beyotime Biotechnology, Shanghai, China) plus 1% phenylmethanesulfonyl fluoride (Beyotime Biotechnology, Shanghai, China). Protein concentration was determined using the Enhanced BCA Protein Assay Kit (Beyotime Biotechnology, Shanghai, China). For proteins of different sizes, equal amounts (20 μg) were separated on 6–12% SDS-PAGE and transferred to a 0.45 pore-size PVDF membrane (Merck KGaA, Darmstadt, Germany). Next, 5% skimmed milk (Beyotime Biotechnology, Shanghai, China) was blocked for 2 h, and the membranes were incubated with specific primary antibodies overnight at 4 °C: BAD, BCL-2, caspase-3, LAMC2, p85, AKT, p-AKT, IκBα, β-action (1:1000 dilution, Proteintech, Rosemont, IL, USA), p-IκBα, p65, p-p65 (1:500 dilution, Proteintech, IL, USA), LAMA3, p-p85 (1:500 dilution, Abways, Shanghai, China), and GAPDH (1:7000 dilution, Abways, Shanghai, China). The membranes were then incubated with secondary antibodies (1:7000 dilution, Proteintech, IL, USA) for 2 h. Finally, an Amersham Imager 680 (GE Healthcare, Sunnyvale, Chicago, IL, USA) was used with a BeyoECL Moon kit (Beyotime Biotechnology, Shanghai, China) for luminescence detection, and protein bands were analyzed using Image J (v1.53c) for results.

### 4.12. Statistical Analyses

In this study, data analysis and graph visualization were conducted using GraphPad 8.0.1 (GraphPad Software, Inc., San Diego, CA, USA). Unpaired *t*-tests were used to assess differences between two groups, and one-way ANOVA with Tukey–Kramer post hoc tests was used to analyze differences between several groups. A *p*-value of less than 0.05 was deemed statistically significant.

## 5. Conclusions

This study reveals a previously unrecognized molecular mechanism by showing that miR-212-5p directly targets *LAMC2* and *LAMA3* and, thus, associates with PM_2.5_-induced apoptosis via the PI3K/AKT/NF-κB pathway. In addition, this is the first study whose findings represent an important breakthrough in understanding cellular damage at the molecular level. These findings not only redefine the role of miR-212-5p in apoptosis, but are also thought to play a major role in identifying highly promising therapeutic targets for combating PM_2.5_-induced lung injury. By shedding light on an entirely new regulatory mechanism, this study sets a new direction for future research and stresses the urgent need for molecular-based solutions to environmental health crises.

## Data Availability

Data is contained within the article and Supplementary Materials.

## Supplementary Materials

The following supporting information can be downloaded at: https://www.mdpi.com/article/10.3390/ijms26041761/s1.
