# Supplementary material for: miR-212-5p Regulates PM2.5-Induced Apoptosis by Targeting LAMC2 and LAMA3
Source: Int J Mol Sci. 2025 Feb 19;26(4):1761. doi: 10.3390/ijms26041761 (PMC11855808; doi:10.3390/ijms26041761)
Supplement: Supplementary file 1 [file ijms-26-01761-s001.zip › ijms-3458000-supplementary.pdf]

**Primer ID**	**Sequences(5′ to 3′)**
miR-212-5p mimics-sense	ACCUUGGCUCUAGACUGCUUACU
miR-212-5p mimics-antisense	UAAGCAGUCUAGAGCCAAGGUUU
miR-mimics NC-sense	UUCUCCGAACGUGUCACGUTT
miR-mimics NC-antisense	ACGUGACACGUUCGGAGAATT
miR-212-5p inhibitor	AGUAAGCAGUCUAGAGCCAAGGU
miR-inhibitor NC	CAGUACUUUUGUGUAGUACAA
siRNA-LAMC2-sense	CCAAACAAGCACUUUCAUUTT
siRNA-LAMC2-antisense	AAUGAAAGUGCUUGUUUGGTT
siRNA-LAMA3-sense	GGCCGUAUCACUGUUUACUTT
siRNA-LAMA3-antisense	AGUAAACAGUGAUACGGCCTT
siRNA-NC-sense	UUCUCCGAACGUGUCACGUTT
siRNA-NC-antisense	ACGUGACACGUUCGGAGAATT

Table S1. The sequence information of mimics, inhibitors, and siRNAs

Primer ID	Sequences(5′ to 3′)	Tm (℃)	PCR program
qPCR-LAMA3-F	AGGAGGTGACAGGGAAGT	60	95℃ 30 sec, (95℃ 5 sec, Tm 60 sec) 40 cycles, 95℃ 5 sec, 60 ℃ 60 sec, 95 ℃ 1 sec, 37 ℃ 30 sec
qPCR-LAMA3-R	CAGGGTGGAAGTTGAAGGAA	60	
qPCR-LAMC2-F	CGAGCAGGCTACTATCATCTG	60	
qPCR-LAMC2-R	AGCTGTCTGAATGCCCATAG	60	
qPCR-GAPDH-F	CCTGCACCACCAACTGCTTA	60	
qPCR-GAPDH-R	CATCACGCCACAGCTTTCCA	60	
RT-miR-212-5p	GTCGTATCCAGTGCAGGGTCCGAGGTATTCGCACTGGATACGACAGTAAG	74	25 ℃ 30 min
qPCR-miR-212-5p-F	CGCGACCTTGGCTCTAGACTG	60	95℃ 5 min, (95℃ 10 sec, Tm 30 sec) 40 cycles, 95℃ 5 sec, 60 ℃ 60 sec, 95 ℃ 1 sec, 37 ℃ 30 sec
qPCR-miR-212-5p-R	AGTGCAGGGTCCGAGGTATT	60	
qPCR-U6-F	CTCGCTTCGGCAGCACA	60	
qPCR-U6-R	AACGCTTCACGAATTTGCGT	60	

Table S2. The sequence information of RT-PCR and RT-qPCR

Primer ID	Sequences(5′ to 3′)	Tm (℃)	PCR program
LAMC2-pmirGLO-WT-F	TCTAGTTGTTTAAACGAGCTCCTCACCTTCATGCTCTCCACCT	61	
LAMC2-pmirGLO-WT-R	CAGGTCGACTCTAGACTCGAGCCCAAAGGAAGACTGGATTAAGAT	63	
LAMC2-mVenus-WT-F	AGTCCGGACTCAGATCTCGAGCTCACCTTCATGCTCTCCACCT	68	
LAMC2-mVenus-WT-R	CAGAATTCGAAGCTTGAGCTCCCCAAAGGAAGACTGGATTAAGAT	68	94°C 3 min, (94 °C 30sec, Tm 30 sec,
LAMA3-pmirGLO-WT-F	TCTAGTTGTTTAAACGAGCTCCCTACACAGCAAGATTCACCTTTG	62	72°C 1 min) 30 cycles, 72 °C 10 min
LAMA3-pmirGLO-WT-R	CAGGTCGACTCTAGACTCGAGCCTTGTGCGAATGTTTGAATGA	62	
LAMA3-mVenus-WT-F	AGTCCGGACTCAGATCTCGAGCCTACACAGCAAGATTCACCTTTG	68	
LAMA3-mVenus-WT-R	CAGAATTCGAAGCTTGAGCTCCCTTGTGCGAATGTTTGAATGA	68	
LAMC2-MUT-F	TACCAGCCAAGGAATCCCTGGGAAAGTATTTCCTTT	65	
LAMC2-MUT-R	GGATTCCTTGGCTGGTAAAAAAAAAAAAAAAATGCTTTC	66	95°C 30 sec, (95 °C 15 sec, Tm 15 sec,
LAMA3-MUT-F	TACCACCAGCCAAGTGTACCCCTCCCCCATCAA	64	72°C 4 min) 30 cycles, 72°C 10min
LAMA3-MUT-R	TACACTTGGCTGGTGGTACTTCCAAAGGTGAAT	64	

Table S3. The sequence information of PCR
